# The Synergy between Zinc and Antimicrobial Peptides: An Insight into Unique Bioinorganic Interactions

**DOI:** 10.3390/molecules28052156

**Published:** 2023-02-25

**Authors:** Caroline Donaghy, Jose Gabriel Javellana, Young-Jin Hong, Karrera Djoko, Alfredo M. Angeles-Boza

**Affiliations:** 1 Department of Chemistry, University of Connecticut, Storrs, CT 06269, USA; 2 Department of Chemistry, Durham University, Durham DH1 3LE, UK; 3 Institute of Materials Sciences, University of Connecticut, Storrs, CT 06269, USA

**Keywords:** antimicrobial peptides, host defense peptides, zinc, metalloAMPs

## Abstract

Antimicrobial peptides (AMPs) are essential components of innate immunity across all species. AMPs have become the focus of attention in recent years, as scientists are addressing antibiotic resistance, a public health crisis that has reached epidemic proportions. This family of peptides represents a promising alternative to current antibiotics due to their broad-spectrum antimicrobial activity and tendency to avoid resistance development. A subfamily of AMPs interacts with metal ions to potentiate antimicrobial effectiveness, and, as such, they have been termed metalloAMPs. In this work, we review the scientific literature on metalloAMPs that enhance their antimicrobial efficacy when combined with the essential metal ion zinc(II). Beyond the role played by Zn(II) as a cofactor in different systems, it is well-known that this metal ion plays an important role in innate immunity. Here, we classify the different types of synergistic interactions between AMPs and Zn(II) into three distinct classes. By better understanding how each class of metalloAMPs uses Zn(II) to potentiate its activity, researchers can begin to exploit these interactions in the development of new antimicrobial agents and accelerate their use as therapeutics.

## 1. Introduction

Zinc(II) ions and antimicrobial peptides (AMPs) are two key players in the innate immune response in most host organisms. Both effectors are found in relatively high concentrations in various cells and tissues. Therefore, one could hypothesize that they not only interact with each other, but that they do so synergistically; that is, a combination of both Zn(II) and an AMP results in an effect that surpasses the individual substances in isolation [1]. Indeed, there are examples in the literature that suggest such a synergistic interaction exists, and it is biologically relevant. However, there is a dearth of understanding of the structural, mechanistic, and biological determinants of the antimicrobial action for this combination of agents. This is not surprising, as this type of mechanistic study requires a highly interdisciplinary approach that weaves fields as divergent as inorganic chemistry and microbiology. In this work, we review the antimicrobial activity of Zn(II) and AMPs alone, and then we highlight examples in which a combination of both effectors leads to an increase in antimicrobial activity.

## 2. Susceptibility of Microorganisms towards Zinc(II) Ions

Zn(II), along with other *d*-block metal ions, plays a major biochemical role in the immune system. This *d*^10^ ion is a cofactor in proteins, where it can play the part of a catalytic center as well as a structural building block [2,3]. A deficiency in Zn(II) has been linked to high susceptibility with bacterial infections [4,5], whereas increased levels of Zn(II) result in the activation of various immune cells [6]. Studies in mammals have shown that during inflammation, elevated concentrations of Zn(II) are found at the sites of infection [7]. In human cells, approximately 10% of cellular Zn(II) is located in the membrane, 50% in the cytoplasmic space, and the remaining is found within the nucleus [8]. Innate immune cells such as neutrophils, which help defend the host against invading microbes through phagocytosis, degranulation, and the release of neutrophil extracellular traps, require Zn(II) to normally function [5]. Neutrophils as well as macrophages have been found to utilize Zn(II) when managing bacterial infections [9,10]. In the vacuoles of bacteria-infected macrophages, Zn(II) concentrations were found to increase after 24 h of infection, although the values varied based on the bacterial strains. In *Mycobacterium tuberculosis*-infected macrophages, the Zn(II) concentration in the phagolysosome at 1 h was found to be in the order of tens of micromolar, a value that increased to hundreds of micromolar after 24 h [11]. This Zn(II) influx suggests that one mechanism utilized by macrophages to inhibit the growth of microbes is to increase Zn(II) ion concentrations. Depending on the microbe, moderate to high concentrations of Zn(II) are needed to inhibit their growth. For instance, the in vitro minimum inhibitory concentration (MIC) values of Zn(II) against a panel of Gram-negative bacteria containing *Escherichia coli*, *Klebsiella pneumoniae*, *Acinetobacter baumannii*, *Pseudomonas aeruginosa*, and *Enterobacter cloacae*, varied between 1–4 mM [12]. It has also been shown that there are increased Zn(II) concentrations at sites of infection in mice, indicating an important role played by Zn(II) in our immune system. On the other hand, Zn(II) is also involved in nutritional immunity, where the concentration and availability of metal ions essential for bacterial growth are reduced at sites of infection. This concept is exemplified by the release of calprotectin in neutrophil extracellular traps, which subsequently binds Zn(II) and lowers the availability of this metal ion at sites of inflammation. The question remains as to how Zn(II) can be utilized in bactericidal or bacteriostatic mechanisms.

Microbes tend to have a moderate tolerance for increased Zn(II) availability [13]. Briefly, let us take a look at how microbes handle Zn(II). For the reader interested in the topic, there are several excellent reviews published within the last decade [5,14,15]. The system for Zn(II) homeostasis in Gram-negative bacteria is perhaps best represented by the system found in *Escherichia coli*. The influx system includes the inducible, high-affinity importer ZnuABC, an ATP-binding cassette (ABC) transporter, and the constitutively expressed ZupT, a member of the ZIP transporter family. The efflux system of Zn(II) is constituted by a cation diffusion facilitator (CDF), ZitB, and a P-type ATPase transporter, ZntA [4,16], both of which are transcriptionally inducible by Zn. In Gram-positive bacteria, exemplified by the streptococci, there is typically one ABC transporter for Zn(II) uptake, AdcBC, which is associated with at least two Zn(II)-binding lipoproteins, AdcAI and AdcAII, and several accessory proteins. A Zn(II) efflux pump, CzcD, has also been identified, which is regulated by a transcriptional activator [4,10,17].

The mechanisms of Zn(II) homeostasis in eukaryotic microbes such as fungi are more complex. These microbes need to transport Zn(II) not only between the extracellular environment and the cytoplasm but also between the cytoplasm and different intracellular organelles. Some fungi species, such as, for example, *Saccharomyces cerevisiae*, *Cryptococcus gattii*, and *Cryptococcus neoformans*, regulate Zn(II) uptake exclusively in a zinc-dependent manner [18]. Meanwhile others, *Aspergillus fumigatus*, *Candida albicans*, and *Candida dubliniensis*, depend on both Zn(II) and pH variances for their Zn(II) uptake regulation [18]. Other fungi species have had their Zn(II) uptake characterized, but with too limited of data to classify them into the stated two mechanisms of regulation [18]. There are two major zinc transporter groups described for fungi, the Zrt/Irt-like protein (ZIP) and CDF families of transporters [18,19]. The regulation of these families depends on the species for which the mechanism is utilized. Within the ZIP family there is Zrt1, Zrt2, and Zrt3, which act as a high affinity transporter, low affinity transporter, and vacuolar exporter, respectively, in most of the fungi species characterized. The CDF has two proteins, Zrg17 and Zrc1, which transport Zn(II) from the cytoplasm into the endoplasmic reticulum (ER) and vacuole, respectively. Each of these transporters are found within the region of genes defined as the zinc responsive elements (ZREs) and are regulated by Zap1, a protein with seven zinc finger domains [19,20].

An excess of Zn(II) ions inside cells can exert a toxic effect on microbes by mismetallating into other metal-binding sites in proteins and enzymes. For example, studies with *Streptococcus pneumoniae* showed that the toxicity of Zn(II) arises from its ability to outcompete Mn(II) ions for binding to the Mn(II) uptake protein, PsaA. The binding of Zn(II) to PsaA results in Mn starvation and increased susceptibility of the bacteria to reactive oxygen species (ROS), likely a result of the loss in the activity of the Mn-dependent superoxide dismutase [21]. Metabolic studies with *S. pyogenes* have suggested that an excess of Zn(II) causes the inhibition of two central glycolytic enzymes, phosphofructokinase and glyceraldehyde-3-phosphate, leading to an impairment of glucose metabolism. In addition, excess Zn(II) also disrupts hyaluronic acid capsule biosynthesis through the inhibition of phosphoglucomutase, thereby also disturbing key virulence pathways [17]. In *E. coli*, Zn(II) overload has been correlated with inhibiting iron-sulfur (Fe-S) cluster biogenesis. Zn(II) is thought to compete with Fe(II) ions or Fe-S clusters for binding to Fe-S cluster assembly proteins [22,23]. In particular, the scaffold protein, IscU, iron chaperone protein, IscA, and the ‘capacitor’ protein, ferredoxin, are the assembly proteins Zn(II) is hypothesized to target. Due to the Zn(II) toxicity mechanism in *E. coli* being linked to Fe-S cluster biogenesis, the preassembled Fe-S clusters are presumed to remain unaffected [22]. In addition, Zn(II) salts can inhibit the SOS response and the hypermutator phenomenon in *E. coli* strains and in *Klebsiella pneumoniae* [24]. Interestingly, Zn(II) acetate was more effective in inhibiting the SOS response than Ni(II), Cu(II), Fe(II), and Mn(II) salts. RecA expression on Shiga-toxigenic *E. coli* was reduced to c.a. 50% when cells were treated with Zn(II) acetate. Interestingly, the use of Zn(II) pyrithione instead of the acetate salt led to an increase in activity of almost two orders of magnitude, indicating that the counterions are not innocent in the activity of Zn(II). We suspect that this is due to the ability of the different Zn(II) salts to penetrate passively into the cells, since cation diffusion through lipid bilayers depends on the anion [25].

Besides its intracellular behavior, experimental data and molecular dynamics simulations show that Zn(II) also affects the membrane of bacterial cells [26,27]. Studies with biomimetic membranes made of dipalmitoylphosphatidylcholine (DPPC) showed that increasing Zn(II) concentrations increased the DPPC bilayer thickness with a maximum at 0.14:1 Zn:DPPC, at which point additional Zn(II) did not have an effect on bilayer thickness [27]. This data is supported by molecular dynamics simulations, where at 1:15 Zn:lipid ratios, membrane thickness increases and area per lipid decreases, corresponding to the lengthening of the membrane and an increased density laterally. A decreased area compressibility modulus was observed at these concentrations, indicating an increased ability for the membrane to be deformed [26]. The observed effects on membrane mimics hint at potential deleterious effects on microbial membrane function; however, studies are needed to demonstrate this hypothesis.

## 3. Antimicrobial Peptides

AMPs, also known as host defense peptides, are short biopolymers that are ubiquitous in nature. These peptides constitute an important component of the innate immune response of diverse host organisms, ranging from simple, single-celled organisms such as bacteria and fungi to complex, multicellular organisms such as vertebrates and plants [28,29,30,31]. These peptides are usually 8 to 50 amino acids long, amphipathic, and in possession of multiple hydrophobic residues [28,32]. Most of the reported AMPs are positively charged, but anionic AMPs are also common [33]. Often AMPs are found to form an α-helical or β-sheet structure when in an environment with cell membranes or membrane mimics, though counter examples also exist. The families of peptides are typically defined by the organisms from which they derive, though they can also be classified according to their structural components, such as having an abundance of a single amino acid or a particular sequence motif [28,32]. The expression of these peptides by host organisms may be constitutive, or inducible by stress signals from an infection or injury. Interestingly, in addition to antimicrobial activity, some AMPs also exhibit antiviral and anticancer properties [34,35].

Given the rising challenge of antibiotic resistance, AMPs have been widely studied for their potential as new age antibiotics for human and veterinary use [28,32,36]. Indeed, several AMPs have already entered the clinic, e.g., Delmitide, Ghrelin, and OP-145 [31]. Here, AMPs show great promise, since resistance to these peptides is rare [28,32]. In addition, the effectiveness of AMPs is highly influenced by environmental factors such as high salt concentrations, pH, and the presence of divalent cations [1,31,37]. These interactions are potentially exploitable in the clinic. It is also important to note that synergy between AMPs and antibiotics has also been observed [1,37].

The large majority of AMPs have been hypothesized to have the ability to permeabilize microbial lipid bilayer membranes, i.e., these peptides create gaps or “pores” which are either stable or transient and which result in membrane protein reorganization, dissipation of transmembrane potential, and/or bacterial lysis [30,35,38]. As the basis of membrane disruption, electrostatic interactions between AMPs and microbial phospholipids have been hypothesized to be the driving force [28]. Pore formation is further sub-classified depending on the mechanism of interaction between the AMP and the cell membrane. The most common pore-forming mechanisms are toroidal, carpet, or barrel–stave pores. Toroidal and barrel–stave pores are transmembrane, with the peptide needing about 22 residues in an α-helix conformation or about 8 residues as a β-sheet to span the length of the membrane [28]. The toroidal mechanism depends on a single or minimal number of AMP molecules to integrate into the membrane and cause a disruption in the membrane curvature that separates lipid tails and polar head groups. In the barrel–stave model, AMPs that have accumulated at the cell surface insert into the membrane in a perpendicular fashion, forming a pore that eventually leads to the leakage of cellular contents or disruption of ion gradients that results in cell death. The carpet model is described as the loss of membrane integrity as AMPs accumulate parallel to the membrane in a high peptide-to-lipid ratio. Upon the peptides accumulating to a certain threshold, the cell membrane will lose structural integrity causing cell content leakage.

Recent studies indicate that some AMPs make incursions into the cytoplasmic space and possess an intracellular target that can impact key cellular functions [12,39,40]. For example, indolicidin targets nucleic acid biosynthesis [41], Bac7 targets protein biosynthesis [42], histatin-5 targets proteases [43,44], and Clav-A at low pH is thought to have nuclease activity. Multiple other mechanisms that play essential roles in cell function and division are also found for other AMPs. These novel mechanisms are potentially exploitable for the development of human and veterinary therapeutics to combat the emergence and spread of antibiotic-resistant traits in bacteria.

## 4. Nexus between Zn(II) and AMPs

To date, only for a few examples of Zn(II)-dependent AMPs (Zn-AMPs) do we have sufficient information to be certain about their mechanism of action, whereas for a handful of other systems, we only know that there is a synergistic interaction between these two effectors. In this latter group, there is an opportunity for researchers to further explore. Moreover, we can predict that additional systems in which AMPs synergize with Zn(II) will be identified in the near future. With this expectation, and in order to better understand the elucidated systems, we would like to systematize the types of interactions that are plausible when both Zn(II) and AMPs fight a common enemy.

**Scheme 1:**
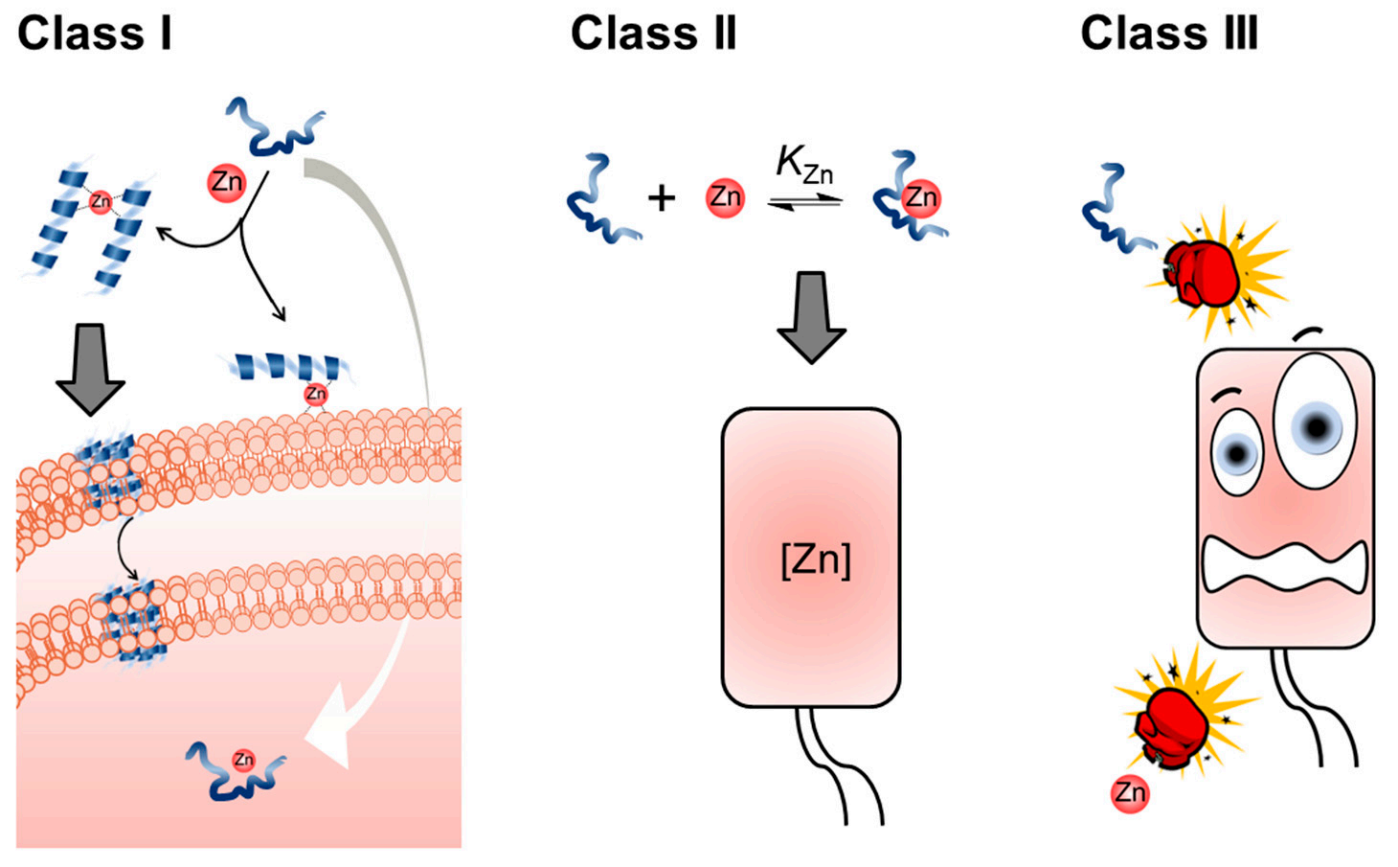
The three different classes of AMP and Zn(II) ion interactions. In Class I, an AMP-Zn(II) complex has a deleterious effect on cells. The Zn(II) complex does not need to be formed by a 1:1 metal ion peptide ratio, the only requisite is that a coordinate bond is formed. In Class II, intracellular Zn(II) availability is controlled by the presence of the AMP. Finally, in Class III, the two antimicrobial effectors do not form a coordination bond at any stage of the process; however, they still help each other resulting in a synergistic effect.

We can think of three broad classes to define the relationships between an AMP and a metal ion like Zn(II). First, in Class I, an AMP activity can be modulated by binding to Zn(II). Here, the metal ion can act as a cofactor that “switches on” (enhances) or “switches off” (suppresses) the antimicrobial activity of the AMP. In a second category, Class II, an AMP can regulate Zn(II) availability. Here, the AMP can participate in host nutritional immunity if it limits the availability of nutrient Zn(II) to the target microbe or increases the concentration of this metal ion inside the pathogen, resulting in toxicity. Finally, in the third scenario, Class III, Zn(II) may influence the activity of an AMP indirectly, without the formation of a Zn(II)-AMP complex. For example, Zn(II) may enhance the activity of a DNA-targeting AMP by inhibiting the SOS response [24]. A depiction of each type of Zn-AMP class can be found in Scheme 1. There are examples to which an AMP will exert multiple MOAs either innately or depending on the environment, so it should be noted that examples of these classes overlapping may exist, but the authors do not address this possibility within this work.

Below, we summarize the literature relating to AMPs whose antimicrobial activities are enhanced by Zn(II) ions. Each AMP and it’s respective interaction with Zn(II) is expanded upon according to past and current literature, and each peptide discussed can be found in Table 1. Most examples belong to the Class I Zn-AMPs, and that is clearly stated in text. In other cases, not enough information is available, and we have preferred not to give a fully distinguished classification, though we hope that the information we provide entices the reader to work on further characterizing these systems.

It is important to note that not all AMPs enhance their antimicrobial activity in the presence of Zn(II). In some AMP-Zn(II) systems, no change in activity is observed, as in the case of clavanin C [45]. For other systems, Zn(II) may suppress the activity of the AMP through mechanisms not yet known, as is observed in the case of KLK8, which gets potentiated by the presence of Ca(II) and Mg(II) ions but losses activity with increasing amounts of Zn(II) [46]. This antagonistic activity could be the direct result of the formation of a complex that precipitates the peptide or the product of a metal-activated cellular pathway that rescue or bypass the toxicity of the AMP. In this review, we exclusively focus on synergistic interactions, although we recognize that the antagonistic interactions can be useful to better understand those systems in which synergy is observed. In the future, the authors hope to broaden this work by expanding on the varying types of interactions with respective examples, uncovering more AMPs that possess positive interactions with Zn(II), unraveling examples where overlap may exist between the types of classes, and distinguishing classifications discussed in this work.

### 4.1. Dermcidin Derived Peptides

Dermcidin (DCD) is a protein composed of 110 amino acids that is expressed by human sweat glands and plays an important role in innate immunity in the skin [47,48]. DCD is proteolytically processed to form several shorter peptide fragments of varying lengths and charges, all of which are released in sweat. The two most abundant DCD-derived peptides are DCD-1 and DCD-1L. DCD-1 is derived from the last 47 amino acids of the C-terminus of DCD, while DCD-1L is identical to DCD-1, with the exception of an additional Leu residue at the N-terminus, which is derived from the parent DCD protein [47,48,49]. Both DCD-1 and DCD-1L have been shown to kill *E. coli*, *E. faecalis*, *S. aureus*, and *C. albicans* in vitro, in both phosphate buffer and a “sweat buffer”, which was prepared to mimic the pH, ionic strength, and sodium, chloride, potassium, and magnesium contents of human sweat [47]. However, while DCD-1L exhibited antimicrobial activity against *E. faecalis* and *S. aureus*, regardless of assay conditions, it showed reduced activity against *E. coli* and *C. albicans* when tested in the “sweat buffer”. By contrast, DCD-1 exhibited strong antimicrobial activity against all tested pathogens, with no loss of activity when tested in the sweat buffer [47]. The antimicrobial activity of peptides under acidic conditions with other metal ions present is minimally explored among AMPs and emphasizes the importance of DCD-derived peptides in innate immunity, as these are the conditions encountered in sweat [47].

DCD-1L has a net charge of -2 at neutral pH. This peptide appears to follow a random coil conformation in aqueous solutions. However, it was observed that an α-helical conformation was induced under various conditions intended to mimic a Gram-negative membrane such as detergents (lauryldimethylamine oxide (LDAO) and n-dodecyl-β-d-maltoside (DDM), phospholipids (diphytanoylphosphatidylcholine (DPhPC), 1-palmitoyl-2-oleoyl-sn-glycero-3-phosphatidylglycerol (POPG), and 1-palmitoyl-2-oleoylphosphatidylethanolamine (POPE), and trifluoroethylene (TFE) [49,50]. It has also been observed that DCD-1L can oligomerize in human sweat in vitro, and the oligomers are stabilized in the presence of Zn(II). This oligomerization of DCD-1L is hypothesized to be crucial to the antibacterial mechanism of DCD-1L, since co-treatment with the non-specific metal chelator EDTA reduces the antibacterial activity of DCD-1L in vitro [49].

**Figure 1:**
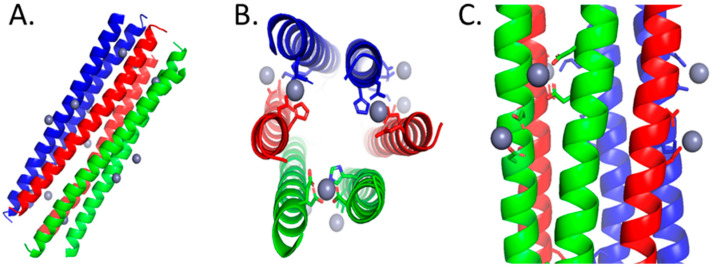
The color coordination signifies the paired peptide helices that are oriented in head-to-tail directions. Amino acids with prime labeling are associated with the peptide in the reverse direction. (**A**) Full-length structure of DCD-1L forming a trimer of Zn-bridged dimers. (**B**) Top-down view of the trimer of dimers highlighting the amino acids, Glu5, Glu9, His38′, and Asp42′, that coordinate to the inner sphere of Zn(II). (**C**) The trimer of dimers’ 3° structure is also stabilized by amino acids, Asp24 & Asp28 and Asp24′ & Asp28′, in the middle of helix binding to Zn(II). All structures were generated in PyMOL [52].

The antibacterial mechanism of DCD-1L has been found to be the formation of ion channels or pores in bacterial membranes, which is a common mechanism for AMPs [49]. It is important to note that the ion channels observed only allowed for the transfer of a limited number of ions at a time, indicating slow kinetics for membrane potential breakdown. The pores were also too small to be observed via electron microscopy or the propidium iodide staining of the bacteria [50]. The formation of pores is supported by correlating the oligomerized structure (Figure 1) elucidated from X-ray diffraction analysis with ss-NMR spectroscopy and molecular dynamics simulations, where DCD-1L was found to form trimers of Zn(II)-bridged dimers in solution containing POPE/POPG, resulting in hexameric channels that resemble a barrel–stave pores in the bacterial membrane [51].

Since Zn(II) was found to be essential for the oligomerization of DCD-1L, this peptide is an example of Class I Zn-AMPs. The metal ion coordinates with the side chains of the N- and C- terminal residues of dimerizing peptides via Glu5 and Glu9 on one monomer and His38′ and Asp42′ on the second monomer [51]. Since the overall Zn(II):DCD-1L ratio is 1:1, Zn(II) also neutralizes the overall charge of the hexameric AMP channel [51]. The amino acids of both monomer units involved in Zn(II) coordination follow the *i*, *i* + 4 motif, which is expected when an alpha helix binds to Zn(II). A variant form of DCD-1L where His38 is substituted by an Ala was found to be incapable of pore formation in vitro, further emphasizing the importance of Zn(II) coordination in the antibacterial mechanism of DCD-1L. However, the effect of this mutation on the antibacterial activity of DCD-1L is yet to be tested [51].

### 4.2. Clavanin A

Clavanins are a family of cationic, His-rich, amphipathic, α-helical peptides isolated from the tunicate, *Styela clava*. Clavanin A (ClavA), is one member of the clavanin family; it contains 23 amino acids and has shown a broad spectrum of antibacterial and antifungal properties while maintaining a low cytotoxicity [53,54]. Unlike other AMPs that have a narrow effective pH, ClavA is active at both acidic (pH 5.5) and physiological (pH 7.4) pH, although it is more active under acidic conditions. ClavA also maintains its antimicrobial activity at high salt concentrations [53]. ClavA was initially hypothesized to exert its antibacterial properties by permeabilizing bacterial membranes at physiological pH (pH 7) [55]. However, the antibacterial mechanism of ClavA has been found to change depending on pH as well as ion availability [12,53,56].

The antibacterial activity of ClavA is enhanced by Zn(II), as evidenced by the up to 16-fold decrease in the MIC of ClavA when tested against an antibiotic-resistant clinical isolate of the Gram-negative bacterium *E. coli* [12,45]. The fractional inhibitory concentration (FIC) of ClavA and Zn(II) against this isolate was 0.14, a value that indicates strong synergy between these two effectors [1,45]. The imidazole sidechains of His17 and His21 in ClavA are hypothesized to be engaged in binding Zn(II). However, no coordination complex between ClavA and Zn(II) has been characterized. This pair of His residues forms an *i*, *i* + 4 motif, similar to the one found in DCD-1L. Mutation of these two residues to Ala residues decreases the antimicrobial activity of the peptide [45].

**Figure 2:**
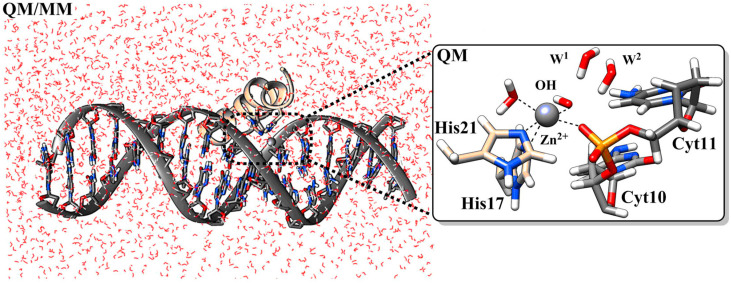
The MD-equilibrated structure of a ClavA-Zn(II) complex showing the initial step in the hydrolysis of DNA. Some hydrogen atoms and solvent water molecules were omitted for clarity. Nitrogen is shown in blue, phosphorus in orange, and oxygen in red. Reprinted with permission from Reference [12]. Copyright 2022, American Chemical Society.

ClavA exhibits three mechanisms of action which depend on the experimental conditions. A combination of bacterial cytological profiling with principal component analysis showed that at physiological pH (pH 7.4), ClavA disrupts bacterial membranes in a manner akin to magainin 2. At acidic pH (pH 5.5), ClavA acts intracellularly by binding DNA or hydrolytically cleaving DNA, as seen in Figure 2, with both mechanisms leading to different phenotypes. The hydrolytic cleavage occurs through a Zn(II) complex formed in cellulo. Interestingly, experiments with *E. coli* mutant strains lacking the high-affinity Zn(II) uptake transporter (ZnuABC) demonstrated that the peptide does not carry Zn(II) into *E. coli* cells. Instead, the peptide appears to use the pool of intracellular Zn(II) ions. Based on this evidence, ClavA can be considered a Class I Zn-AMP when acting at pH 5.5.

### 4.3. Bacitracin

Bacitracin is a cyclic peptide antibiotic isolated as a mixture of closely related dodecapeptides from *Bacillus subtilis* and *Bacillus licheniformis*, and it has been widely studied since its discovery in 1945. It is used as a preventive drug for livestock and as a component in the “triple antibiotic” ointments Neosporin and Polysporin [57,58]. Bacitracin A, the major component of Bacitracin, possesses the strongest potency against bacteria [59]. However, Bacitracin only has narrow spectrum activity against Gram-positive bacteria, and minimal activity against Gram-negative bacteria. The antibiotic activity of Bacitracin is dependent upon the presence of divalent metal ions, including Zn(II) [60,61].

Bacitracin is a Class I Zn-AMP, in that the peptide exerts antibacterial activity when bound to a divalent metal such as Zn(II). This AMP acts by inhibiting bacterial cell wall synthesis. The Zn(II)-AMP was found to bind with undecaprenyl pyrophosphate, a carrier lipids responsible for shuttling cell wall biosynthesis intermediates [58]. The binding of Bacitracin with the lipid carrier forms a 1:1:1 lipid:peptide:metal complex which results in an incomplete synthesis of the cell wall, ultimately leading to the hindrance of bacterial growth [61,62]. The formation of the peptide–lipid complex was observed to be pH-dependent, with the lowest dissociation constant being approximately 1 × 10^−6^ M, or 1 µM, between pH 7–7.5, with significantly higher constants outside that range. As the ionizable pyrophosphate group of the lipid is likely deprotonated under all conditions tested (the pKa of pyrophosphate is expected to be 1.77 under aqueous conditions), the effect of the pH is likely due to the ionization state of the bacitracin peptide. The dissociation constant for the formation of a peptide–metal–lipid complex between the lipids and the metal-bound peptide was also investigated for different metal ions. Binding to Zn(II) was found to result in the highest affinity when compared with other divalent metals, such as Cd(II) and Mg(II) [63]. However, it is important to note that the lipid used in this experiment was inorganic phosphate rather than undecaprenyl pyrophosphate. This was done since the inorganic phosphate had the lowest initial binding affinity to the peptide, and thus would yield the largest change in affinity due to the addition of a metal ion.

**Figure 3:**
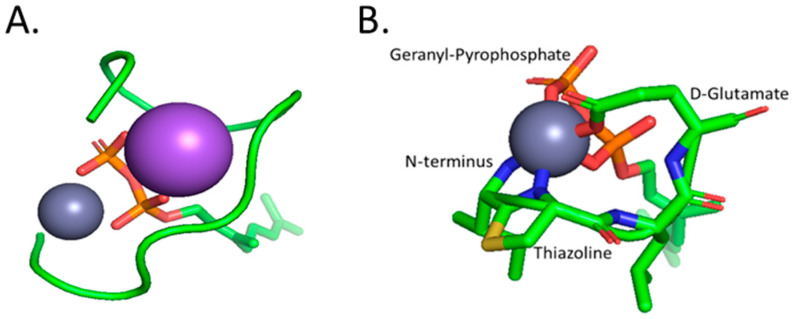
(**A**) The full complex of Zinc coordination with the geranyl-pyrophosphate ligand, represented in red (oxygen), orange (phosphorus), and green for the geranyl chain, and bacitracin displayed in a green ribbon representation. Sodium and Zinc are represented as the large purple sphere and small grey sphere, respectively. The sodium ion found within the complex is thought to neutralize the pyrophosphate charge and bridge oxygen atoms from the pyrophosphate and C-terminal of Bacitracin. (**B**) A zoomed-in depiction of the inner-sphere coordination of Zinc with the N-terminal amine, amino acids, D-Glu4 and thiazoline, oxygens from the geranyl-pyrophosphate, and an unrepresented water molecule. All structures were generated in PyMOL [52].

The crystal structure of the Bacitracin-Zn(II)-lipid complex shows the peptide wrapping around the pyrophosphate of the lipid and the Zn(II) ion [58]. The orientation of Bacitracin around the pyrophosphate almost completely shields the pyrophosphate from the environment, with only 2% of the pyrophosphate’s surface area remaining solvent-accessible. The complex was also found to adopt an amphipathic configuration, which is likely to support association with the lipid membrane. In addition to a Zn(II) ion, the curved structure formed by Bacitracin also contains a sodium ion [58]. The previously unreported sodium ion is hypothesized to both neutralize the negative charge of the pyrophosphate and to bridge two of the oxygen atoms of the pyrophosphate with oxygen atoms from the C-terminal of the AMP. Zn(II), on the other hand, was observed to coordinate with the peptide at three points, namely the amino terminus, Glu-4, and the nitrogen of thiazoline. In addition, Zn(II) also coordinates with two oxygen atoms from the pyrophosphate group and a water molecule (Figure 3) [58]. This coordination is unique relative to the other AMPs described here, as it assumes an octahedral geometry rather than a tetrahedral geometry. as Additionally, the Zn(II) coordination does not involve any His side chains, which have been shown to be essential in Zn(II) coordination by other AMPs.

### 4.4. Kappacin

Kappacin is an anionic antimicrobial peptide originating as a nonglycosylated, phosphorylated fraction of the bovine milk protein, κ-casein(residues 106–169) [64]. Kappacin is effective against *Streptococcus mutans* with an MIC of 0.68 mg/mL [64]. It was found that Kappacin increases the permeability of synthetic liposomes at pH 6.5 but not at pH 7.2, indicating that its mode of action is pH-dependent and membranolytic in nature [65]. ^1^H-NMR spectroscopy found that the addition of excess calcium ions was able to change the secondary structure of the active region of Kappacin (residues 33–53) from a random coil to an alternative conformation which may correspond to an α-helix, though further analysis is needed to confirm this [65].

When tested against *S. mutans* biofilms, Zn(II) was able to increase the antibiofilm effects of Kappacin, with a 1:2 Kappacin:Zn(II) ratio corresponding to the highest efficacy [65]. Scatchard analysis confirmed that purified Kappacin contained two Zn(II) binding sites [65]. Unlike the aforementioned AMPs, Kappacin does not contain any His residues [66]. Instead, the active region of Kappacin contains several Glu residues which have been involved in Zn(II) coordination in both Bacitracin and DCD-1L. The Glu residues in the active region also follow the *i*, *i*+4 pattern at three separate instances, which can explain the increased α-helicity in the presence of divalent ions. Overall, although Zn(II)-peptide complex formation is a possible explanation for the synergy between Kappacin and Zn(II), which would make it a Class I Zn-AMP, further testing is needed to confirm the coordination and interactions between Zn(II) and Kappacin.

### 4.5. Calcitermin

Calcitermin is a 15 amino acid C-terminal cleavage of the larger antimicrobial protein, calgranulin C (CaGC). The parent protein belongs to the S100 family of proteins, known to bind to Cu(II). Calgranulin C is mainly produced by neutrophils and monocytes and is thought to play a role in inflammatory host defense [67,68]. Calcitermin was first isolated from human nasal secretions [67]. The antimicrobial activity of calcitermin was tested against *E. coli*, *E. faecalis*, *P. aeruginosa*, *C. albicans*, *S. epidermidis*, *S. aureus*, and *L. monocytogenes*. At neutral pH, calcitermin was found to exert no antimicrobial activity against any of the tested pathogens. However, under acidic conditions, calcitermin was found to exert an activity against *E. coli*, *E faecalis*, *P. aeruginosa,* and *C. albicans* [67,69]. The acidic pH is likely more physiologically relevant, as the pH of inflammatory liquid, in which calcitermin is found, is often acidic [67,69]. When tested using the radial diffusion assay, it was observed that, at a high Zn(II) concentration (100 μM), Zn(II) was able to increase the activity of calcitermin against *E. coli*, while at low concentrations, there was no significant difference [67]. Interestingly, in the presence of Zn(II) at high concentrations, calcitermin was found to also be active against *L. monocytogenes,* even though it was previously not active against this organism in the absence of Zn(II).

Analysis through NMR, ESI-MS, and potentiometry suggest that the coordination sphere of Zn(II) with calcitermin utilizes residues His9, His11, His13, and Glu15. Though the C-terminal carboxyl group instead of the Glu15 cannot be fully excluded from this coordination, though comparative studies with other His-rich AMPs suggested that, under acidic conditions, the Glu15 residue further stabilized the Zn(II)-calcitermin system [69]. The same study that better elucidated the coordination between calcitermin and Zn(II) also found that residues, His9 and His11, play a more critical role in the coordination of Zn(II) compared to the His13 through mutational studies [69]. The synergistic bioactivity of calcitermin with Zn(II) in some cases has been found to be more active than common antibiotics against *C. albicans*, *E. faecalis*, and *S. aureus*, though the patterns of this synergy remain to be elucidated. An example of this complex synergy, though His9 is thought to be pivotal in Zn(II) binding, the bioactivity of calcitermin with an H9A mutation was found to be more active against *C. albicans* compared to the wild type in the presence and absence of Zn(II) [69]. At this stage, calcitermin will be classified as a Class I Zn-AMP, but more structural and biochemical characterization is needed for greater confidence in this designation.

### 4.6. *ARVA Peptide

The *ARVA peptide is a 12-amino-acid, cationic synthetic peptide designed to have a β-sheet secondary structure in membranes. The sequence of the peptide is RRGWALRLVLAY-NH_2_, and it was selected from a combinatorial peptide library based on its ability to permeabilize membranes [70]. It was observed that *ARVA is active against a broad spectrum of microbes, including Gram-positive *S. aureus*, Gram-negative *E. coli*, and *P. aeruginosa*, and the fungi *C. albicans* while maintaining a low toxicity towards mammalian cells [70,71]. *ARVA shows synergistic behavior with metal ions against some of the previously mentioned pathogens [71]. It was found that *ARVA is synergistic with Zn(II) against *P. aeruginosa* (FIC = 0.43) and *S. aureus* (FIC = 0.30) while being additive against *E. coli* (FIC = 0.71) and *C. albicans* (FIC = 1.34).

**Figure 4:**
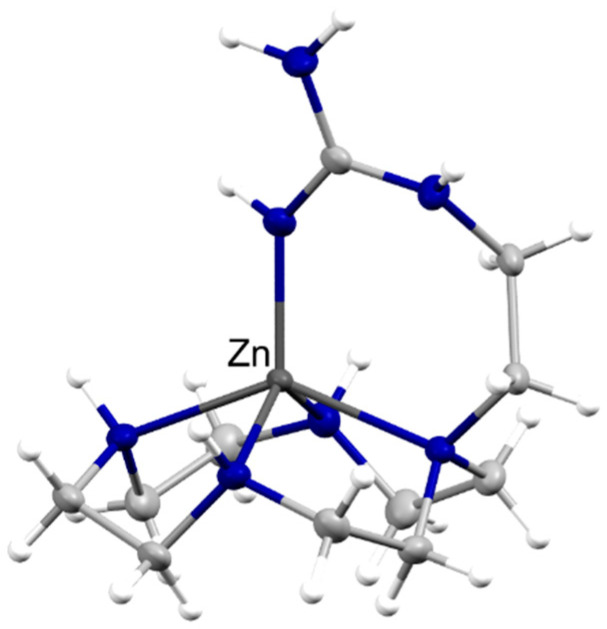
Arginine can be a good ligand to Zn(II). Thermal ellipsoid plot at 50% probability of a Zn(II) complex of the ligand (2-guanidinyl)ethyl-cyclen (CSD code XIZZOV). The complex was formed in an aqueous solution at neutral pH. The two perchlorate anions have been omitted for clarity. The zinc atom is labeled, whereas the nitrogen atoms are depicted in blue.

Similar to Kappacin, *ARVA does not contain any His residues, which is uncommon in the AMPs with extensively studied synergy with Zn(II). Rather than increasing the stability of the secondary structure of *ARVA by metal coordination, Walkenhorst et al. hypothesized that Zn(II) exhibits synergy with *ARVA due to the ability of *ARVA to increase membrane permeability for small ions [71], which would make it an example of a Class III Zn-AMP. It is interesting to note that Zn(II) binding to guanidinium functional groups in arginine residues is possible in neutral pH aqueous solution (*I* = 0.1) [72], as seen in Figure 4; however, it is likely that other functional groups in the peptide should have a high affinity for Zn(II) for binding to be biologically relevant.

### 4.7. Histatins

Of all of the AMPs described in this review, histatins are perhaps the most extensively studied. Histatins comprise a family of structurally related, cationic, His-rich antimicrobial peptides found in humans and other higher primates. These peptides are all derived from two parent genes, *HTN1* and *HTN3* [73]. These peptides were first identified in saliva, but recent work has shown that they are also found in human tears.

Upon secretion by the salivary glands, the two parent histatins, namely Histatin-1 (Hst1) and Histatin-3 (Hst3), are processed proteolytically by unidentified salivary proteases or proteases from resident oral microbes into shorter fragments, which include the major fragment Histatin-5 (Hst5) [74]. Hst1 (38 aa), Hst3 (32 aa), and Hst5 (24 aa) and represent the major histatins, accounting for nearly 90% of all histatins isolated from fresh salivary gland secretions [75]. The concentrations of these full- and longer-length fragments in whole saliva decrease dramatically, a result of further proteolysis. Thus far, at least 24 histatin fragments have been identified in whole saliva [76]. It has been found that Histatins have a concentration of around 50 μg/mL upon secretion from parotid glands; Hst5, being the major component, had a concentration between 15–30 μg/mL in whole saliva [77]. This concentration range should be considered by researchers attempting to elucidate the role of Hst5 within oral cavities and oropharynx.

Histatins are thought to play an innate immune role in the human oral cavity by contributing to the defense against potentially harmful microbes. In addition, Hst1 also promotes wound healing by activating epithelial cell migration [78]. Hst5 is known to display bactericidal and fungicidal properties in vitro against many of the ESKAPE (*Enterococcus faecium*, *Staphylococcus aureus*, *Klebsiella pneumoniae*, *Acinetobacter baumanni*, *Pseudomonas aeruginosa*, and *Enterobacter species*) pathogens [79] and members of the *Candida* genus, including *C. albicans*, *C. kefyr*, *C. krusei*, and *C. parapsilosis* [80,81,82]. It is worth noting that the antibacterial and antifungal activities of Hst5 were observed only in dilute-salt buffers that do not mimic the ionic strength of saliva. In high-salt buffers, these activities are abolished.

Hst5 is only weakly amphipathic and is intrinsically disordered in aqueous solutions [74]. Nevertheless, it can fold into a more helical structure in a membrane-like environment. Unlike many AMPs, Hst5 is not thought to disrupt fungal membranes, although it does disrupt the membranes of Gram-negative bacteria [79], possibly as a side effect of the actual MOA. Hst5 is generally thought to enter the cytoplasm in an energy-dependent manner of target microbes, for example via non-specific, membrane-bound polyamine transporters, Dur3 and Dur31, in *C. albicans*. Once inside the cytoplasm, Hst5 it is thought to cause cell death through the generation of reactive oxygen species, alterations in mitochondrial functions, and non-lytic potassium and ATP efflux [44,77,80].

Hst5 contains two potential Zn(II) binding sites, HxxxHH and HExHH [83,84], that bind Zn(II) with high micromolar dissociation constants. Both sites follow the *i*, *i* + 4 motif that is also present in DCD-1L and ClavA. It is thought that Hst5 binds to Zn(II) with a 2:2 Zn(II):Hst5 stoichiometry [84], in which each of the Zn(II) ions coordinates to four His residues, two each from both Hst5monomers, thus forming a Zn(II)-bridged dimer. This binding geometry is similar to that proposed for DCD-1L, as described earlier. Binding of Zn(II) to Hst5 appears to stabilize the peptide [83,84]. As would be expected from the role of His side chains in coordinating Zn(II), the binding is observed only at neutral and basic pH in which the imidazole side chains are deprotonated [84,85].

It has been shown that Hst5 candidacidal activity increases in the presence of Cu(II) and decreases in the presence of Fe(III), while the relationship between Zn(II) and Hst5 has been difficult to define due to widely conflicting results reported in the literature [44]. However, several recent lines of evidence suggest that Zn(II) mitigates the bioactivity of Hst5 in a stoichiometric-dependent manner. At low Zn(II):Hst5 ratios of 0.5:1 or less, Zn(II) was shown to enhance the antifungal activity of Hst5 against *C. albicans* [44,80]. In fact, fungal cells that were treated with Hst5 and sub-stoichiometric concentrations of Zn(II) were shown to be less virulent when compared with untreated cells, a result of Zn(II)-induced reorganization of the fungal cell wall and impaired adhesion to host epithelial tissues [80]. By contrast, at Zn(II):Hst5 ratios of 1:1 or higher, Zn(II) was shown to suppress the antifungal activity of Hst5 against *C. albicans*. Fluorescence imaging further showed that co-treatment with stoichiometric or super-stoichiometric concentrations of Zn(II) inhibited the uptake of Hst5 into the fungal cytoplasm [44].

The effects of Zn(II) on the antibacterial activity of Hst5 are less defined. A recent preprint indicates that Hst5 does not influence growth or survival of multiple *Streptococcus* species that typically colonize the oral cavity and thus interact with salivary fluids [86], while the addition of Zn(II) neither enhanced nor suppressed this effect. However, a similar study by these authors did observe a rescuing effects from Hst5 for a streptococcal strain with impaired Zn(II) efflux within a buffer that mimicked saliva [86]. Reviewing the literature as a whole suggests that Hst5 may have a greater role in developing the microbial composition within an oral cavity by exerting selective antimicrobial activity and maintaining commensal forms of the microbiota, possibly being dictated through signaling of Zn(II) concentrations [44,86,87].

Given the relatively weak affinity of Hst5 to Zn(II) within biologically relevant environments, it has been argued that a Zn(II)-Hst5 complex is not likely to form in vivo or even in in vitro conditions in which potential competing Zn(II) ligands such as phosphates or amino acids are present [86]. In addition, it was recently shown that Hst5 does not promote Zn(II) starvation in *Streptococcus* due to its inability to compete for binding Zn(II) with the AdcAI Zn(II) uptake protein [86]. Therefore, Hst5 is not a Class II Zn-AMP. Altogether, it seems that Hst5 could act as either a Class I or III Zn-AMP. Further studies will need to confirm this classification.

For the readers interested in working with the histatins, the formation of histatin-Zn(II) complexes can be used to purify these AMPs from parotid saliva. Flora et al. demonstrated that treatment of this parotid secretion with ZnCl_2_ at pH 9 allows recovery of up to 90% histatins (Hst1, Hst3, and Hst5), as revealed by a combination of cationic PAGE, HPLC, and mass spectrometry [88]. Subsequent elution through a desalting column should allow the recovery of *apo* peptide [88].

### 4.8. Shepherin

Shepherin I and II (ShepI and ShepII) are AMPs first isolated from the roots of *Capsella bursa-pastoris*, commonly known as shepherd’s purse [89]. Both of these peptides are Gly- and His-rich and are found to contain a Gly-Gly-His repeating motif in their sequences [89,90]. Alone, ShepI and II exhibited activity against multiple Gram-negative bacteria with IC50 values in the low µg/mL range against *E. coli*, *P. putida*, *P. syringae*, and *Serratia sp.* These peptides also showed activity against fungi, with IC50 values ranging from 2.5 to 8 µg/mL against *C. albicans*, *C. neoformans*, and *S. cerevisiae* [89]. CD analysis in 50% TFE showed that the majority of the secondary structure for both peptides is random coils, with a β-sheet character detected in Shep I (33%) and no α-helices detected in either peptide [89].

Shep I and some of its derivatives showed synergistic activity with Zn(II) against a variety of *Candida* species [90,91]. Shep I and the Shep I C-terminal amidated analogues, Shep Ia(amidated), Shep I(3-28)a, and Shep I(6-28)a, tested increased their activity up to 8-fold in the presence of 10 µM ZnCl_2_ [90]. In the case of Shep Ia, the increase in activity was not observed when 10 µM Ca^2+^, Mg^2+^, or Cu^2+^ was present, indicating that the synergy is unique to Zn(II) [91]. Additional studies showed that a fluorescein-labeled version of Shep Ia (FAM-Shep Ia) is internalized into *Candida* species via a direct translocation as well as endocytosis. FAM-Shep Ia is found in both the vacuoles and cytoplasmic space of *Candida* cells. When extracellular Zn(II) was added, a decrease in the number of cells containing green fluorescence was observed. This result initially suggest that peptide internalization is inhibited by Zn(II). However, a separate experiment without cells present showed that the fluorescence of FAM-Shep Ia is quenched by the presence of Zn(II). Thus, an alternative explanation for the observed fluorescence quenching is possible: intracellular FAM-Shep Ia may bind to intracellular Zn(II) ions. Certainly, additional studies are needed to discriminate between the two scenarios. The fluorescence quenching results further suggest that, since the peptide was labeled at the N-terminus, the Zn(II) binding site is likely located close to that end of the peptide, given that quenching of fluorescence by metal ions such as Zn(II) is sensitive to distance [92]. The synergy between Shepherin and Zn(II) can likely be classified as a Class I Zn-AMP interaction, but again more data is needed to confirm this classification.

### 4.9. Surfactant-Associated Anionic Peptides (SAAPs)

SAAPs are a family of peptides isolated from the ovine pulmonary surfactant preparations. They are Asp rich, anionic, and have selective bioactivity exclusively in the presence of Zn(II) [93]. These peptides, SAAP2 (GDDDDDD), SAAP3 (DDDDDDD), and SAAP6 (GADDDDD), are termed based off their fraction label from HPLC purification of the supernatant of ovine pulmonary surfactant suspension [94]. Phosphate inhibits their bactericidal activity, while Ca(II) and Mg(II) were found to have no effect. Zn(II) is required in the antimicrobial activity against *K. pneumoniae*, *E. coli*, *S. aureus*, *S. faecalis*, *Pasteurella haemolytica*, and *Mannheimia haemolytica* [93,95]. Though, only *P. haemolytica* and *Mannheimia* had MIC values in the low micromolar range [93,95]. The bioactivity of synthetic aspartic acid polymers was also tested, and it was observed that the MIC of these peptides was proportional to the number of Asp residues and that Zn(II) was still required for their bactericidal functionality [93].

As stated above, Zn(II) is a cofactor in the bioactivity of all the SAAPs, though the MOA of the SAAPs and the synergistic relationship between the AMPs and Zn(II) is yet to be elucidated. Recent work using ITC measurements determined that SAAP2, SAAP3, and SAAP6 bind Zn(II) in a net-entropically driven process at buffer-independent binding constants of 1.4 × 10^5^, 1.3 × 10^5^, and 2.5 × 10^5^, respectively, at pH 7.4 [95]. These values indicate that the three SAAPs bind Zn(II)with similar affinity. The ITC measurements, along with potentiometry and mass spectroscopy, have led to the hypothesis that the N-terminus amine, Asp1, Asp2, and water molecule are responsible for the coordination sphere between Zn(II) and SAAP3 [95].

Interestingly, the SAAPs were found to not disturb microbial membranes, but instead evidence shows intracellular damage [93,95]. Due to the SAAPs likely having an unidentified intracellular target and having the capability to bind Zn(II), the synergistic relationship is difficult to distinguish among the three proposed classes. It is possible that the SAAPs utilize the Zn(II) to reduce their charge or to stabilize the structure for interaction with the intracellular target, which would make it a Class I Zn-AMP, but further research is needed to make a classification.

## 5. Concluding Remarks

Both Zn(II) ions and AMPs are essential components of the immune system. Despite the fact that both effectors encounter each other when fighting pathogens, very little attention has been paid to the effects one has on the other. In this work, we have attempted to review the literature that corresponds to natural and synthetically derived AMPs that enhance their antimicrobial activity in the presence of Zn(II) ions. Any omissions are a product of our inadequate review of the literature and not because we think that there is little scientific value in that work. We propose a classification for this novel family of peptides: Class I, corresponding to AMPs that form a complex with Zn(II); Class II, in which members regulate zinc availability; and Class III, in which Zn(II) and AMPs act independently but enhance the activity of their companion. It should be kept in mind that overlap among the different classes are potentially possible (maybe the Histatins are the first example), since many antimicrobial agents act using more than one mechanism of action.

Besides the intrinsic beauty of these systems, there is a utilitarian side to this research, as AMPs are a class of antibiotics that are already having an impact in the clinic [31]. A new generation of antimicrobial agents that take advantage of the host pool of Zn(II) ions, or other antimicrobial effectors, will allow our society to battle more efficiently against pathogens, including antibiotic-resistant bacteria. The recent work identifying that Hst5 and Zn(II) help maintain *C. albicans* as a commensal microbe hints at a larger function of Zn-AMPs, and probably metalloAMPs in general, as shapers of microbiota composition and maintainers of homeostasis [28]. Since understanding how one can regulate the relationship between commensal bacteria, host, and pathogen will lead to the development of safe and effective therapeutics against pathogens, more studies on the role of Zn-AMPs are needed.

## Figures and Tables

**Table 1:** Each of the metalloAMPs we focused on in the different families discussed are organized in this table defining their sequence, structural features when within environments that mimic membranes, and their Zn-AMP classification. The sequences that included D-amino acids are designated by their one-letter code being in bold, and the cyclized portion designated within the sequence. Peptides with amidated C-terminals are indicated with an amine included at the end of the sequence. Classifications that need further research to achieve full designation are indicated with an asterisk.

Peptide	Sequence	Structural Features in Membrane Mimetic Environments	Classification
DCD-1L	SSLLEKGLDGAKKAVGGLGKLGKDAVEDLESVGKGAVHDVKDVLDSVL	α-helical, Oligomerizes to trimers of Zn(II)-bridged dimers in presence of Zn(II)	Class I
Clavanin A	VFQFLGKIIHHVGNFVHGFSHVF-NH_2_	α-helical	Class I
Bacitracin	ICL**E**I-cyclo(K**O**I**F**H**D**N)	Cyclic peptide, Mixture of dodecapeptides	Class I
Kappacin (33-53)	AVESTVATLEDSPEVIESPPE	Slight α-helix upon binding to divalent ion	Class I *
Calcitermin	VAIALKAAHYHTHKE	α-helical	Class I *
*ARVA peptide	RRGWALRLVLAY-NH_2_	β-sheet	Class III
Histatin-5	DSHAKRHHGYKRKFHEKHHSHRGY	Slightly α-helical, Forms oligomers upon interaction with Zn(II)	Class I and/or III
Shepherin I	GTGGHGGHGGHGGHGGHGGHGHGGGGHG	Slight β-sheet	Unclassified
Shepherin II	GYHGGHGGHGGGYNGGGGHGGHGGGYNGGGHHGGGGHG	Random coil	Unclassified
SAAP2	GDDDDDD	Random coil	Unclassified
SAAP3	DDDDDDD	Random coil	Unclassified
SAAP6	GADDDDD	Random coil	Unclassified
